# Network Traffic Prediction Incorporating Prior Knowledge for an Intelligent Network

**DOI:** 10.3390/s22072674

**Published:** 2022-03-30

**Authors:** Chengsheng Pan, Yuyue Wang, Huaifeng Shi, Jianfeng Shi, Ren Cai

**Affiliations:** 1 School of Electronics and Information Engineering, Nanjing University of Information Science & Technology, Nanjing 210044, China; 20201249336@nuist.edu.cn (Y.W.); shihuaifeng@nuist.edu.cn (H.S.); jianfeng.shi@nuist.edu.cn (J.S.); 20201218004@nuist.edu.cn (R.C.); 2 School of Automation, Nanjing University of Science and Technology, Nanjing 210044, China; 3 National Mobile Communications Research Laboratory, Southeast University, Nanjing 210044, China

**Keywords:** network traffic prediction, self-similarity, Hurst exponent, a priori knowledge, intelligent networks

## Abstract

Network traffic prediction is an important tool for the management and control of IoT, and timely and accurate traffic prediction models play a crucial role in improving the IoT service quality. The degree of burstiness in intelligent network traffic is high, which creates problems for prediction. To address the problem faced by traditional statistical models, which cannot effectively extract traffic features when dealing with inadequate sample data, in addition to the poor interpretability of deep models, this paper proposes a prediction model (fusion prior knowledge network) that incorporates prior knowledge into the neural network training process. The model takes the self-similarity of network traffic as a priori knowledge, incorporates it into the gating mechanism of the long short-term memory neural network, and combines a one-dimensional convolutional neural network with an attention mechanism to extract the temporal features of the traffic sequence. The experiments show that the model can better recover the characteristics of the original data. Compared with the traditional prediction model, the proposed model can better describe the trend of network traffic. In addition, the model produces an interpretable prediction result with an absolute correction factor of 76.4%, which is at least 10% better than the traditional statistical model.

## 1. Introduction

With the spark of Industry 4.0, the IoT has witnessed huge development in recent years in our daily life, such as smart homes, smart cities, smart stores, and smart buildings. The IoT makes our lives easier; however, ubiquitous IoT devices, such as smart phones, create a huge amount of data every day, which requires large computing resources for analysis, creating significant challenges. Rapid technological development, the increasing number of terminals, the richness of multimedia applications, and the continuous expansion of network capacity are continually increasing consumer demand for Internet resources; however, the service quality of the network is facing significant challenges in meeting this demand. Unlike traditional networks, intelligent networks are green and ubiquitous. Key nodes in such networks are equipped with computing and storage capabilities, and the characteristics of the network traffic change before and after the network traffic passes through these key nodes. In an intelligent network, accurate and effective prediction can allow for an understanding of the network traffic characteristics in advance, which can be used to improve network resource utilization and prevent network congestion [1,2]. Therefore, it is especially important to establish an efficient and reliable prediction model for network traffic.

The essence of network traffic prediction is time-series forecasting (i.e., to build a function of the characteristics of the nodes to be predicted, concerning time variation, based on historical data). Common network traffic prediction models can be divided into two major categories: linear prediction and non-linear prediction. Linear prediction methods mainly use a polynomial fitting function to approximate the actual network traffic, and the fitting effect is improved when there are a large number of parameter adjustments. The traditional linear forecasting models are the historical average model [3], autoregressive model, autoregressive sliding average (also known as the autoregressive moving average model), and modified models based on these [4,5]. However, linear prediction methods have difficulty in capturing non-linear features, such as rapid fluctuations, and the time-dependence of network traffic. Non-linear prediction techniques have emerged with the introduction of artificial neural networks [6,7,8], for example, data-driven deep learning models, such as convolutional neural networks (CNNs) [9] and recurrent neural networks (RNNs) [10,11], in addition to machine learning algorithms, such as support vector regression (SVR) [12] and Transformer [13].

**Figure 1:**
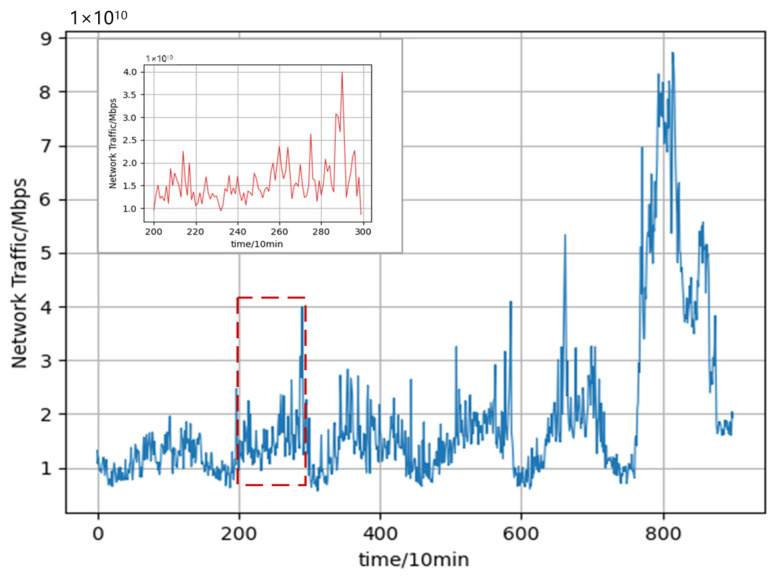
Self-similarity of network traffic.

Although neural network-based models have demonstrated impressive predictive performances, deep learning models are often treated as black-box models. Compared with traditional statistical models, the learning process of deep learning algorithms is intricate and less interpretable. Existing studies have shown that the network traffic flow has obvious self-similarity [14]. In other words, the local and overall time-series have a certain connection, in which the shape of a part of the series is very similar to the overall series, and the network traffic time-series shows similar abrupt changes at different time scales over a long period. Figure 1 depicts the obvious self-similarity of network traffic. It shows the change in the traffic within a week while the magnified inset shows the change in the traffic within one day, from which it can be seen that the trend of the network traffic change in a week is roughly the same as that in a day; that is to say, the historical traffic data and the data to be predicted have the same characteristics and, thus, this similarity can be used to enhance the accuracy and interpretability of network traffic prediction.

To explore the accuracy and interpretability deeply, this paper proposes a network traffic prediction model, named FPK-Net (Fusion Prior Knowledge Network), which incorporates prior knowledge. The self-similar characteristics of traffic are considered as prior knowledge for prediction, which is added to the learning process of the neural network model, thus increasing the interpretability of the model. The main contributions of this paper are summarized as follows.

1. FPK-Net consists of a CNN and an LSTM based on an attention mechanism. The self-similarity property is incorporated into the training model before model training, which results in improvements in the extraction of traffic features and in the prediction accuracy when dealing with insufficient sample data.
2. The model incorporates the self-similarity property of network traffic as a priori knowledge into the intermediate structure of the deep network; namely, the Hurst exponent is added into the gating of the long short-term memory neural network (LSTM) as a bias term to increase the model’s interpretability.
3. Experiments on publicly available datasets verified that the proposed model is consistent with the existing empirical evidence, and has better predictive power than other existing prediction methods. The accumulation of prior knowledge during training meaningfully guides the network traffic prediction, thus significantly improving the performance of the training model. In terms of the absolute coefficient of correction, the proposed model achieved values at least 10% higher than those of traditional statistical models. Thus, the reliability and superiority of the proposed model were illustrated in the article while the results demonstrated that the model is also interpretable.

## 2. Related Work

Existing time-series forecasting models can be divided into two main categories: linear forecasting models and non-linear forecasting models. Of these, linear forecasting uses traditional statistical methods and can be applied to the prediction of smooth series. Common linear forecasting models are the historical average (HA) [3], autoregressive moving average [4] (ARMA), and autoregressive integrated moving average model (ARIMA) models [5]. The historical average (HA) model uses historical averages as predictions [15] while the ARMA model, developed by P.H. Zou et al., can be used to predict the feasibility of exceeding the threshold of network traffic. Rishabh et al. [16] decomposed the network traffic data into linear and non-linear components based on a discrete wavelet transform (DWT), and then used the autoregressive integrated moving average (ARIMA) for the prediction of non-linear components. At present, however, the burstiness of smart network traffic is high and traditional models, such as Poisson distribution modeling, are only suitable for predicting small network traffic sequences, being unable to effectively extract the characteristics of network traffic at different scales in different times. Thus, they cannot meet the characteristics of smart network traffic.

With the development of artificial intelligence, many machine learning and deep learning models have been used to predict network traffic, and such non-linear prediction models have shown good prediction results on non-stationary sequences. For example, Lei et al. [17] proposed the use of wavelet neural networks and artificial neural networks to predict decomposed traffic sequences; however, this approach relies on the selection of wavelet basis functions and the initialization of parameters. Z. Yang et al. [18] proposed a multi-stage prediction model using the grey wolf optimization algorithm and a support vector machine (GWO-SVR), but this approach generally relies on the selection of a kernel function. Extreme learning machine (ELM) [19] and ELM combined with a decomposed fruit-fly optimization algorithm (FOA-ELM) have been used to predict the low- and high-frequency components after traffic decomposition; however, ELM models are generally less stable and improvement of their accuracy depends on an increase in the number of hidden nodes in the network. Num et al. proposed a long short-term memory (LSTM) model to predict network traffic sequences. LSTM is a variant of RNN, which can overcome the disadvantage of gradient disappearance inherent to RNN models, and which has exhibited an efficient non-linear time-series modeling capability; however, as the length of the prediction sequence increases, it is difficult for a single LSTM network to converge to the global optimum, and the learning process of deep learning algorithms is opaque and less interpretable, compared to traditional statistical models. Wu N et al. [13] proposed the prediction of network traffic with Transformer. However, such deep learning models lack interpretability, and the Transformer is poor at establishing long-term dependency capture when long time series need to be predicted.

At present, there are two mainstream algorithms for fusing prior knowledge: Adarsh P. [20] proposed the model-driven approach using prior knowledge for pre-processing to improve machine learning algorithms; however, this approach is overly reliant on artificially determined hyper-parameters and is prone to missing information during conversion. It is not wise to completely disregard prior knowledge when extracting features from deep learning models. Xie Y [21] proposed the model-driven approach using prior knowledge as the intermediate structure in deep networks [22], which can effectively fuse prior knowledge and data by using the prior knowledge to change part of the structure of the network. For example, R. Ramachandran [23] proposed the fractional autoregressive integrated moving average (FARIMA) model, whereby the parameter *d* in FARIMA(*p*,*d*,*q*) can be obtained. When using FARIMA for prediction, the Hurst exponent of the time-series must first be identified. Furthermore, to predict the future network traffic size, the Hurst exponent, which portrays the self-similarity property, is introduced into the prediction algorithm; however, the ARIMA model itself has limitations, and its prediction effect is poor and not suitable for non-linear prediction.

## 3. A Network Traffic Prediction Framework Incorporating Prior Knowledge

### 3.1. Problem Definition

The problem of predicting network traffic in a single-feature time-series scenario can be described as follows:

- A time series is a node v, which only has one feature $X_{t}$ at a given moment t.
- The prediction problem refers to the information that is used to predict the next moment $X_{t+1}$ from historical data $\left(X_{1},X_{2},\ldots ,X_{t}\right)$, where $S_{1,\ldots ,t}$ is the relevant characteristic obtained from the historical data $\left(X_{1},X_{2},\ldots ,X_{t}\right)$; that is, to find the information F(.) that satisfies:
  (1) $$X_{t+1}=F(X_{1},X_{2},\ldots ,X_{t}|S_{1,\ldots ,t})$$

### 3.2. Prediction Framework

**Figure 2:**
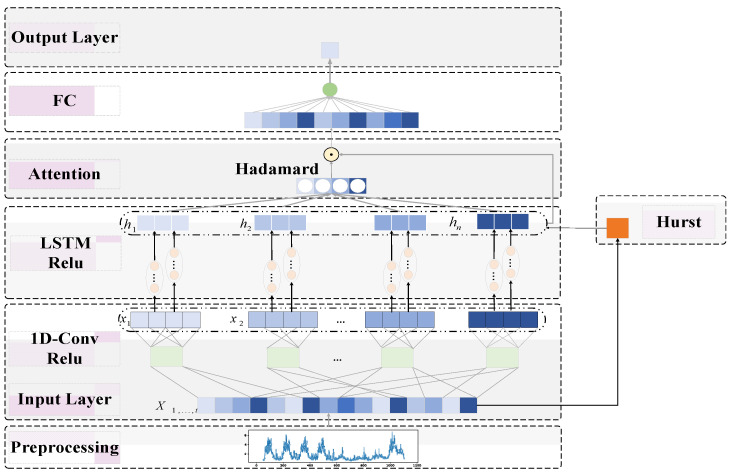
Network traffic prediction framework incorporating prior knowledge (FPK-Net).

The network traffic prediction model with fused prior knowledge, which we call FPK-Net, consists of three main components: a traffic characterization module, a traffic feature extraction module, and a fused prior knowledge module. Among them, the flow characterization module consists of an R/S analysis to calculate the Hurst exponent, which is used to measure the self-similarity of flow sequences with different step sizes. The flow feature extraction module consists of one-dimensional convolution to extract the features of the flow sequences, and the fused prior knowledge module takes the parameters derived in the flow characterization module and incorporates them into the long short-term memory gating mechanism. The LSTM extracts coarse-grained features from the fine-grained features extracted from the front-end, refines the processing of different dimensional features to a certain extent, and can avoid memory loss and gradient dispersion caused by an excessively long step length. When the CNN is combined with LSTM, the short-term features of the time-series are ignored; thus, an attention mechanism is added to the CNN–LSTM model to expand the perceptual field of the input and perceive information before and after the time-series comprehensively. The attention mechanism improves the influence of the temporal features while reducing the influence of unimportant features in the model. The CNN–LSTM model based on the attention mechanism is used to fuse coarse- and fine-grained features to comprehensively portray the temporal data. Figure 2 illustrates the general framework of the fused prior knowledge network traffic prediction model (FPK-Net) proposed in this paper.

## 4. Predictive Models

### 4.1. Flow Characterization Module

Realistic network traffic series are usually non-stationary, although these traffic series tend to have obvious self-similarity. The self-similarity property is reflected in the fact that the local and overall time-series are related. Traditional network traffic models are only applicable to small-scale bursty traffic, but the variation characteristics of network traffic data have some similarities under large-scale conditions. Self-similar traffic characteristics differ from short correlated traffic characteristics in that they can reflect similar abrupt changes in the network traffic time-series at different time scales over long periods. The Hurst exponent is based on the asymptotic process of a rescaling range, defined as a function of the period of the time-series, and is used to describe the self-similarity of time-series with long correlation properties, defined as follows: For a given series of length *n*, the sample mean is, the sample variance is, and we have:

(2) $$\frac{R}{S}(n)=\frac{1}{s(n)}[\max(0,W_{1},W_{2},\ldots ,W_{n})-\min(0,W_{1},W_{2},\ldots ,W_{n})]$$

As n→∞, we have $E(\frac{R}{S}(n))=cn^{H}$, where *c* is a constant, and the above equation yields the Hurst exponent H. In particular, *R* refers to the range of the deviations from the mean, *S* to the sum of standard deviations or variances over the sequence, and *W* to the cumulative sums.

After calculation of the Hurst exponent, it can be seen that the time-series shows trends of averaging, regression, aggregation, and so on. The Hurst exponent can be used to measure the long-term memory and fractality of the time-series. As few assumptions are made about the underlying system, the Hurst exponent has wide applicability in time-series. The value of the Hurst exponent can be used to classify time-series trends according to three features: The higher the value, the smoother, less volatile, and less rough the time-series. When its value is between 0 and 1, the time-series has different properties.

When 0<H<0.5, the time-series shows a negative correlation trend, and the time series has violent fluctuations.

When 0.5<H<1, the time-series shows correlation over a long period, which indicates that the network business flow is correlated over time.

When H=0.5, the time-series shows the process of Brownian motion, and the correlation coefficients between the series are 0 and independent of each other.

There are several methods for calculating the Hurst exponent and, in this paper, we choose the rescaling range analysis method to measure the network traffic data. Let a time-series $\left\{X_{1},X_{2},X_{3},\ldots ,X_{N}\right\}$ of length N be divided equally into several shorter time-series, with length on the order of n=N,N/2,N/4. The average readjustment range for each sub-sequence is then calculated as follows:

- Calculate the average value of each subsequence $m=\frac{1}{n}\sum_{i=1}^{n}X_{i}$.
- Create mean-adjusted series $Y_{t}=X_{t}-m,t=1,2,\ldots ,n$.
- Generate cumulative deviation series $z_{t}=\sum_{i=1}^{t}Y_{i},t=1,2,\ldots ,n$
- Calculation range $R_{t}=\max(Z_{1},Z_{2},\ldots ,Z_{t})-\min(Z_{1},Z_{2},\ldots ,Z_{t}),t=1,2,\ldots ,n$
- Calculation range $S_{t}=\sqrt{\frac{1}{t}\sum_{i=1}^{t}{\left(X_{i}-u\right)}^{2}},t=1,2,\ldots ,n$
- Calculating the rescaling range ${\left(R/S\right)}_{t}=R_{t}/S_{t},t=1,2,\ldots ,n$

By taking the logarithm of both sides of the equation, we can obtain the relationship ${\left(R/S\right)}_{t}=\log c+H\log t$, where c is a constant. By plotting the relationship (R/S) with t on the logarithmic axis and depicting the points, all points (logt,log(R/S)) can be found to lie on an almost straight line; therefore, the slope of the regression line can be approximated by the Hurst exponent.

### 4.2. Flow Feature Extraction Module

Traffic feature extraction is one of the key steps in network traffic prediction. A traditional neural network consists of a three-layer structure with input, hidden, and output layers and, although it can extract features and map from features to values, it faces the problem of requiring a large number of parameters. In this paper, we use a multi-layer feed-forward neural network structure (i.e., a CNN), which adds a feature learning part to the traditional neural network and selects features by means of the network itself. Convolutional neural networks are characterized by weight sharing, translation invariance, and local connectivity, allowing for a higher level and more abstract representation of the raw data. By removing noise and unstable components from the data, the local features of the network traffic can be effectively captured. Although CNNs have achieved great success in the field of image processing (i.e., considering two-dimensional data), one-dimensional (1D) data are suitable for processing time-series, such as time series for speech recognition [24], stock prediction [25], etc.

**Figure 3:**
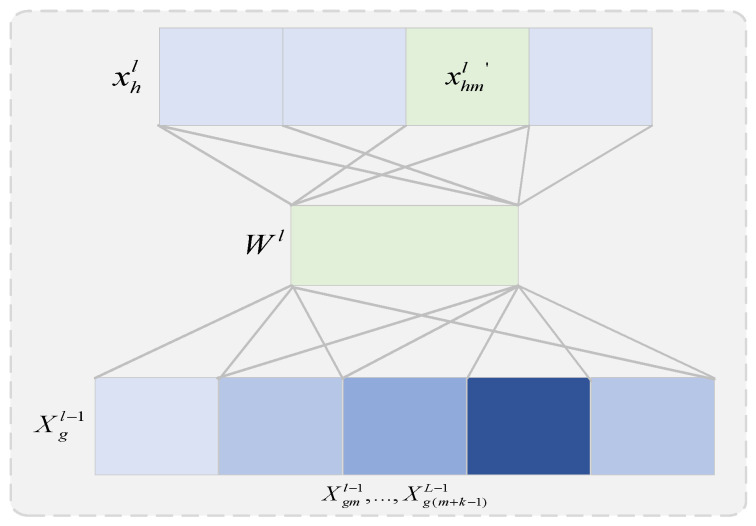
Schematic diagram of one-dimensional convolution.

Network traffic size is a one-dimensional sequence that varies over time and can be represented using a 1×N matrix. One-dimensional convolutional neural networks have a convolutional kernel size of 1×K and use M convolutional kernels to compute sliding over 1×N data, mapping the original traffic data 1×N to a high-dimensional feature space (M×N) and providing a good feature embedding for subsequent capture of temporal features. Traditional neural networks use spatial relativity to reduce the number of parameters and solve the problem of a large number of neural network parameters. Figure 3 shows the 1D convolution process for the hth feature of the lth layer and the *g*th feature of the layer, where, for the 1D convolution kernel, L is the feature length, and the *g*th feature of the (l − 1)th layer can be expressed as:

(3) $$X_{g}^{l-1}=(X_{g1}^{l-1},\ldots ,X_{gm}^{l-1},\ldots ,X_{gL}^{l-1})$$

where ∗ is the convolution operation, $b^{l}$ is the bias, and k is the size of the convolution kernel, which can be obtained by nonlinear activation:

(4) $$x_{hm}^{l}{}^{\prime }=f(W^{l}*X_{g(m,m+k-1)}^{l-1}+b^{l})$$

If s is the number of convolution kernels, the lth feature of the *h*th layer can be written as:

(5) $$x_{h}^{l}=(x_{h1}^{l}{}^{\prime },\ldots ,x_{hm}^{l}{}^{\prime },\ldots ,x_{hs}^{l}{}^{\prime })$$

### 4.3. Integration of Prior Knowledge Modules

**Figure 4:**
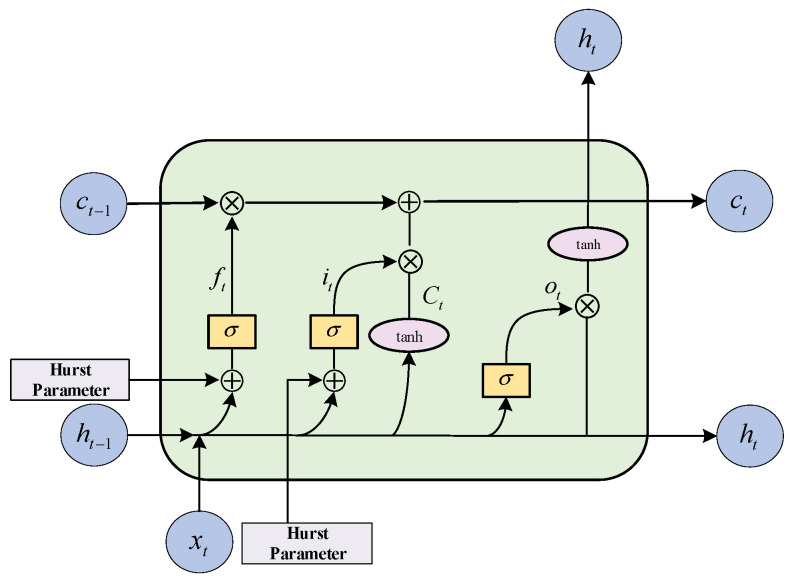
Schematic diagram of the LSTM structure after fusing prior knowledge.

The LSTM model consists of an input layer, a recursive layer (with memory blocks, instead of traditional neuron nodes, as the basic units), and an output layer. A memory block is a set of cyclically connected subnets, each containing one or more self-connected memory cells and three multiplication units—input gates $i_{t}$, output gates $o_{t}$, and forget gates $f_{t}$—which perform continuous simulation of write, read, and reset operations for the cells. The main purpose of the LSTM is to model long-term dependencies and to determine the optimal input length through the use of the three multiplication units. The implicit state of the LSTM is a tuple consisting of two states $\left(c_{i-1},h_{i-1}\right)$. In the initialization state, the tuple is an all-zero tensor, the input tensor at each time is $x_{t}$, and the output tensor is $y_{t}$. As shown in Figure 4, the forget gate $f_{t}$ controls what information needs to be discarded from state $c_{t-1}$ of the previous moment. Thus, it ignores irrelevant features and automatically determines the best input. The input gate $i_{t}$ determines the state that the unit needs to update and, therefore, has a long-term memory capability. The output gate $i_{t}$ filters the output based on the state of the unit. In Figure 4, the LSTM network forward propagation is calculated as follows:

(6) $$f_{t}=\sigma (W_{i}{}_{f}x_{t}+b_{if}+W_{hf}h_{t-1}+b_{hf})$$

(7) $$i_{t}=\sigma (W_{ii}x_{t}+b_{ii}+W_{hi}h_{t-1}+b_{hi})$$

(8) $$g_{t}=\tanh(W_{ig}x_{t}+b_{ig}+W_{hg}h_{t-1}+b_{hg})$$

(9) $$o_{t}=\sigma (W_{io}x_{t}+b_{io}+W_{ho}h_{t-1}+b_{ho})$$

where $h_{t}$ denotes the output vector; w denotes the weight matrix before the linear transformation; $g_{t}$ updates the new information; the symbols ⊗ and ⊕ denote element-level multiplication and element-level concatenation, respectively; σ is the sigmoid function; and tanh is the hyperbolic tangent function.

The traditional LSTM requires the value $f_{t}$ calculated by the sigmoid function to control the flow of the implied state from the previous step $c_{i-1}$ into the next step before calculating the next implied state $c_{i}$. FPK-Net adds the Hurst exponent of the input sequence before the function transformation to improve the forget and input gates of the LSTM. The specific forget and retention values of the LSTM are controlled by the current input and the previous implicit state through the sigmoid function, such that this improved gating mechanism can be considered reasonable. From Equation (6) above, we can see that both the forget gate and input gate finally need to go through the sigmoid function output, and the output takes values between 0 and 1. When the output tends to 1, it indicates a memory state and, vice versa, when it tends to 0, it indicates a forget state. The Hurst exponent reflects the local and overall scale invariance of the traffic sequence; that is, when 0.5<H<1, the trend of the traffic sequence in the future time peri-od can be predicted. The larger the Hurst exponent, the higher the degree of self-similarity, which is positively correlated with the operation mechanism of the forget and input gates. Therefore, if the Hurst exponent is added before the linear transformation, it can meaningfully learn the linear transformation weights and, thus, guide traffic sequence prediction. The improved forget gate and input gate equations are as follows:

(10) $$f_{t}=\sigma [(W_{i}{}_{f}x_{t}+b_{if}+W_{hf}h_{t-1}+b_{hf}+Hurst)]$$

(11) $$i_{t}=\sigma (W_{ii}x_{t}+b_{ii}+W_{hi}h_{t-1}+b_{hi}+Hurst)$$

(12) $$c_{t}=f_{t}\times c_{t-1}+i_{t}\times g_{t}$$

(13) $$y_{t}=h_{t}=o_{t}\times \tanh c_{t}$$

While introducing the information of the previous step, it is also necessary to calculate the information of the current time step $g_{t}$, which is a linear transformation of the tanh activation function combined with the current input tensor $x_{t}$ and the implicit state of the previous step $h_{t-1}$. The amount of information flowing to the neural network needs to be controlled during the calculation, which is obtained by combining the linear transformation of the input tensor and the implied state of the previous step by the product of $i_{t}$ and $g_{t}$, then combining the implied information of the previous step to obtain the information of the new implied state $c_{t}$, as shown above. Finally, the new implied state, $h_{t}$, is calculated using the activation function. The result, $o_{t}$, is obtained by multiplying the sigmoid function with the linear transformation of the input tensor $x_{t}$ and the implied state $h_{t-1}$ from the previous step, and combining the new implied state $c_{t}$ with the output result $y_{t}$. The weight coefficients for the linear transformations vary throughout the computation process. 

The attention mechanism is a weighting of global input features over a space or channel by weights trained by a neural network, so that attention can be obtained for the purpose of focusing on a specific region or channel. The most central operation of the attention mechanism is to train a string of weight parameters, i.e., the importance of each element, through the neural network, and then assign attention to the elements according to their importance. When the CNN is combined with LSTM, the short-term features of the time-series are ignored; thus, an attention mechanism is added to the CNN–LSTM model to expand the perceptual field of the input and perceive information before and after the time-series comprehensively. The attention mechanism improves the influence of the temporal features while reducing the influence of unimportant features in the model. The CNN–LSTM model based on the attention mechanism is used to fuse coarse- and fine-grained features to comprehensively portray the temporal data and improve the accuracy of prediction.

For the final hidden output $h_{t}$ of the long short-term memory neural network, which will be used as the input of the attention layer and requires calculation of the scores corresponding to the different outputs according to their weights, the calculation formula is as follows, where the softmax function is used to calculate a score for the output of the hidden layer to obtain a normalized weight:

(14) $$score=softmax(wh_{t}+b)$$

(15) $$A_{out}=score⊙h_{t}$$

## 5. Experiments and Analysis of Results 

### 5.1. Experimental Data

The dataset used in this paper is the traffic generated by the transit link of the Japanese WIDE network (AS2500) since February 2013, generated through a monitoring tool called Agurim [26]. Agurim is a network traffic monitor based on flexible multidimensional traffic aggregation, which allows users to dynamically switch views at different temporal and spatial granularities depending on the number of flows and packets, addresses, or protocol attributes. The views are dynamically switched, and the supported data sources are pcap, sFlow, and netFlow [27], making the dataset real-time and self-similar.

**Figure 5:**
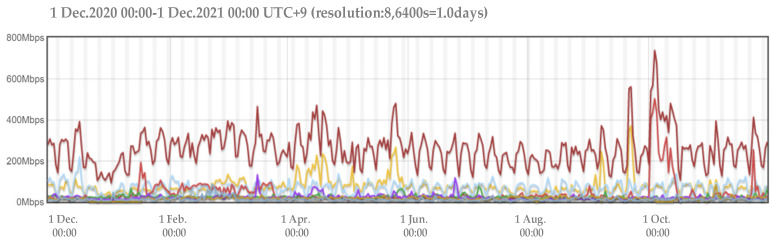
Flow diagram for BPS perspective in Agurim.

The main view in Agurim contains two plots, the first based on BPS (bits per second, i.e., how many bits are sent per second) as shown in Figure 5 and the second based on PPS (packet per second, i.e., how many packets are sent per second). By default, each graph shows seven significant aggregated flows, with the legend labels showing the main attributes of each aggregated flow and their proportion of the total traffic, and the sub-attributes of the aggregated flow and their proportion. In this experiment, the total network flow is aggregated using seven aggregated flows, which are located in the last column of the dataset and therefore do not distinguish between the primary and secondary attributes of the address and protocol. The output data format is a text format based on the BPS pivot view.

In this paper, a total of 52,493 data units were sampled from this data set between 1 January 2020, and 31 December 2020, with a sampling interval of 10 min. For uncontrollable reasons, the sampled data set contained some missing values. As such, we used the zero-fill method to fill in the gaps. The first 70% of the processed data were classified as the training set, 20% as the validation set, and the last 10% as the test set.

### 5.2. Experimental Parameters and Evaluation Metrics

**Figure 6:**
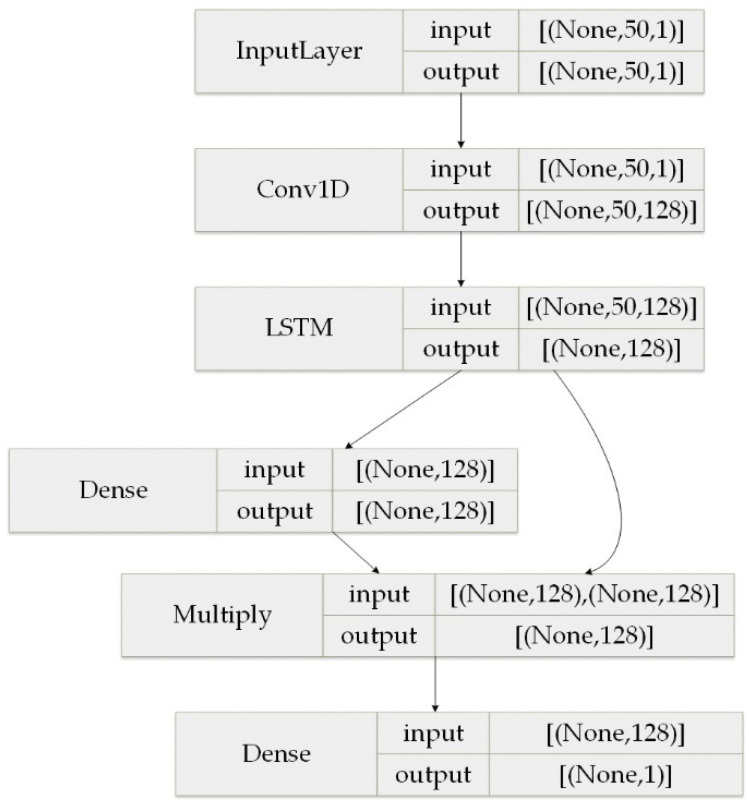
Model structure parameter configuration.

In this experiment, a deep learning server was used to configure the experimental environment, where the CPU production type was AMDRyzen 52,600 and the memory size was 16 GB. In addition, Pytorch was used to build the network framework and Python was the programming environment. In the experiments, the optimizer was selected as the SGD optimizer, with outstanding speed in late iteration. The learning rate was set to 0.001, hidden_size was set to 128, batch_size was set to 64, and the number of training epochs was 20. The detailed model structure parameters were configured as shown in Figure 6. 

When training the model, the L2 norm was used as the loss function, and a regularization term was added to prevent overfitting. The formula is as follows:

(16) $$loss=||Y_{i}-{\hat{Y}}_{i}|{|}_{2}+\lambda L_{reg}$$

where $Y_{i}$ denotes the true value, ${\hat{Y}}_{i}$ denotes the predicted value, λ is the hyper-parameter, and $L_{reg}$ is the canonical term. To verify the validity of the model, common methods used in the field of traffic prediction were selected for experimental comparison, including four algorithms: historical average (HA), autoregressive integrated moving average (ARIMA), support vector machine (SVM), and long short-term memory (LSTM). These were evaluated using four common serial predictors, with $y_{i}$ denoting the true value and ${\bar{y}}_{i}$ denoting the predicted value.

(1) Squared absolute error *(MAE*): This indicator measures the mean absolute error between the error and the true value, taking values in the range of [0,+∞); the closer the MAE is to 0, the better the performance of the model:
  (17) $$MAE=\frac{1}{m}\sum_{i=1}^{m}\left|y_{i}-\bar{y_{i}}\right|$$
(2) Mean square error (*MSE*): This indicator reflects the prediction error of the model, taking a value in a range of [0,+∞); the smaller the error, the better the model performance:
  (18) $$MSE=\frac{1}{m}\sum_{i=1}^{m}{\left(y_{i}-{\bar{y}}_{i}\right)}^{2}$$
(3) The root mean square error (*RMSE*): This indicator reflects the prediction error of the model, taking a value in the range of [0,+∞); the smaller the error, the better the model performance:
  (19) $$RMSE=\sqrt{\frac{1}{m}\sum_{i=1}^{m}{\left(y_{i}-{\bar{y}}_{i}\right)}^{2}}$$
(4) The absolute coefficient of correction ($R_{adapted}^{2}$): This indicator reflects the quality of the model fit, taking values in the range [0,1]; the closer to 1, the better the model performance. Here, m is the total number of samples and p is the number of features:
  (20) $$R_{adapted}^{2}=1-\frac{\left(1-R^{2})(m-1\right)}{m-p-1}$$

### 5.3. Experimental Results and Analysis 

#### 5.3.1. Results of the FPK-Net Model Compared with Other Baseline Models

FPK-Net was compared with the above five methods on the experimental data set. Table 1 shows the results of predicting the network traffic in a future time period.

**Figure 7:**
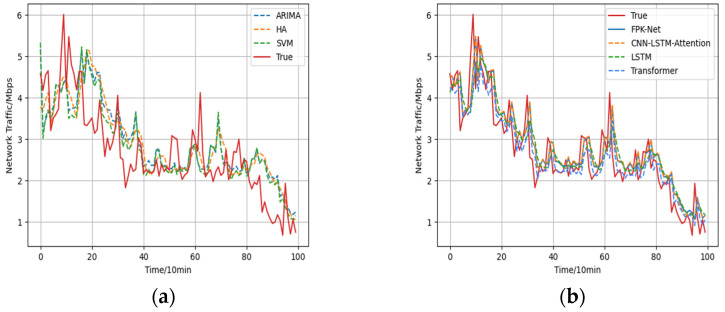
Comparison of prediction results and true values: (**a**) Prediction results of traditional prediction methods vs. true values; (**b**) Prediction results of deep learning models vs. true values.

From the evaluation indicators of each model in Figure 7, Table 1, it can be seen that:

(1) The historical average model (HA) uses the average historical value for forecasting and, in this paper, the average value of the last eight steps was used to forecast the next step. For this method, the forecast error was large.
(2) Due to the limitations of model building, traditional time-series models do not have satisfactory prediction results. Among them, the ARIMA model had the largest prediction error among the above 6 types of models, with MAE and RMSE of 0.615 and 0.757, respectively, and the smallest prediction accuracy, with an $R_{adapted}^{2}$ value of 0.509. As the essence of the ARIMA model is to capture the linear relationship of the flow series without considering the influence of other factors, the ARIMA model had a lesser effective prediction effect.
(3) The support vector machine model (SVM) had the advantages of using fewer training parameters and producing more accurate results. The prediction results were 0.391 for MAE, 0.583 for RMSE, and 0.677 for $R_{adapted}^{2}$. Its prediction results were more accurate than those of traditional statistical methods.
(4) The $R_{adapted}^{2}$ of the LSTM model was 0.750, which indicates that it produced more accurate results than the linear prediction methods. Although the LSTM-based prediction was good and it has a certain degree of feature mining ability for long time-series, as the input series contained more information, it was difficult for the LSTM model to converge to the global optimum during training, which led to poor prediction results.
(5) The transformer model uses a self-focus mechanism to model traffic sequences. The prediction results were 0.412 for MAE, 0.565 for RMSE, and 0.711 for $R_{adapted}^{2}$. Its prediction results were more accurate than other linear prediction methods. Although the transformer forecasts are good, the transformer is less capable of establishing long-term dependence capturing when long time series need to be predicted.
(6) Compared with the other 5 models, the proposed FPK-Net model achieved the best results, in terms of all 4 evaluation indices, and the absolute coefficient of correction of the FPK-Net model reached 76.9% while the root-mean-square error reached 0.509. Compared with the ARIMA model, the RMSE and $R_{adapted}^{2}$ were decreased by 0.248 and improved by 26.0% through the use of FPK-Net, respectively. Meanwhile, compared with the SVM, FPK-Net improved the $R_{adapted}^{2}$ value by 9.2%; the SVM was less effective in prediction as it used a linear kernel function.

Overall, the analysis indicated the poor fitting ability of HA and ARIMA for unstable data and long time-series while the neural network models fit the non-linear data better.

#### 5.3.2. Ablation Experiments

**Figure 8:**
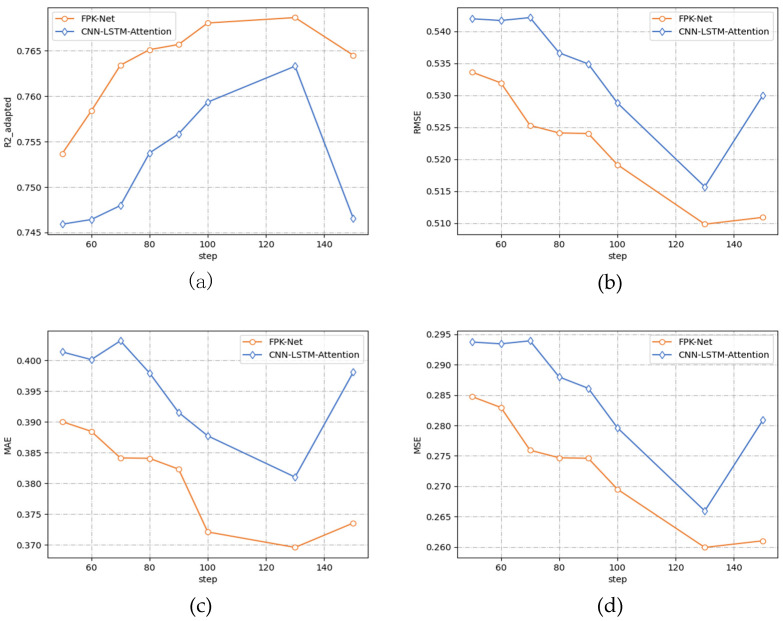
Performance comparison using different values for the sliding window. (**a**) Performance ($R_{adapted}^{2}$) comparison using different values for the sliding window. (**b**) Performance (*RMSE*) comparison using different values for the sliding window. (**c**) Performance (*MAE*) comparison using different values for the sliding window. (**d**) Performance (*MSE*) comparison using different values for the sliding window.

To verify the advanced nature of the FPK-Net model, this section compares the 2 methods CNN+LSTM+Attention and the fused prior knowledge (FPK-Net model) through ablation experiments, where the prediction time step is varied from 50 to 150 min, and the prediction results are shown in Figure 8 below. 

From the above Table 2, it can be seen that:

(1) After adding the Hurst module into the LSTM network, the change trend of the four measures on the two models was consistent. Along with a gradual increase in the size of the prediction step, all three error indicators decreased to a minimum value and then increased while the absolute coefficient of correction increased to a maximum value and then decreased, and the prediction accuracy gradually increased. The error curve presented a concave function while the absolute coefficient of correction presented a convex function.
(2) When the prediction time step reached 130 min, the prediction accuracy reached its peak, and the error was the lowest. At this time, the FPK-Net model had the best prediction effect, with an MAE of 0.369 and MSE of 0.259. When the prediction time step exceeded 130 min and continued to increase, the performance of both models decreased.
(3) From Figure 6 above, it can be seen that the performance of the FPK-Net model proposed in this paper was always better than that of the baseline model, regardless of the time step. In particular, the FPK-Net model, which incorporates prior knowledge, showed the most significant improvement when the step size reached 70 min, with a 1.9% reduction in the MAE measure and a 2.1% improvement in the $R_{adapted}^{2}$ measure.

#### 5.3.3. Interpretability Analysis

**Figure 9:**
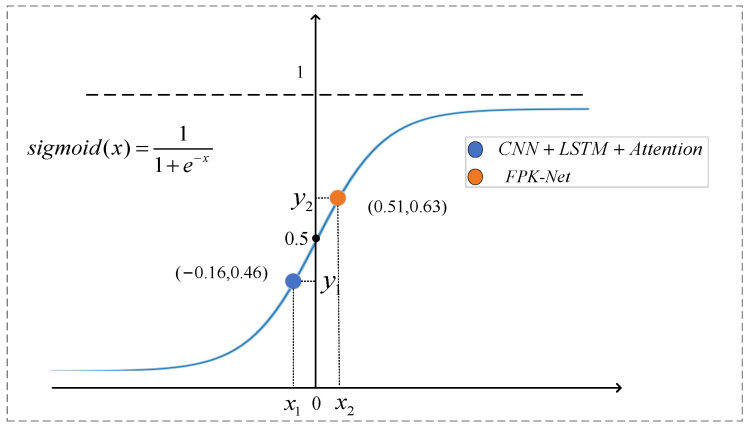
Schematic diagram of interpretability analysis.

As the Hurst exponent provides a measure of predictability, the self-similarity of the time-series corresponding to different time steps varies. The larger the Hurst exponent, the more the value of mapping on the sigmoid function tends to 1. The specific forget and retention values of the LSTM are controlled by the current input and the implicit state of the previous step through the sigmoid function, and the forget gate is in the memory state. As shown in Figure 8 above, when the step size was 70, the output value of the forget gate was 0.46, which is the corresponding value in the figure, and the value of the sequence was 0.67. With the Hurst exponent was added before the linear transformation, the value after linear transformation was closer to 1 (which is the corresponding point in Figure 9). From the above, it can be seen that, when using this value to guide the model training before prediction, the FPK-Net model had the best prediction when the step size was 70. Therefore, we believe that, at this time, by incorporating prior knowledge, the LSTM can efficiently and meaningfully forget some input information from the previous step, and the network traffic sequence with larger exponents can be calculated before attempting to build the prediction model. In addition, one can also focus on sequences with large Hurst exponents, as network traffic with long time scales has self-similarities that can be regularly found, which can save time and effort while allowing for more accurate predictions.

## 6. Conclusions

In this paper, we proposed a traffic prediction method, called FPK-Net, which utilizes fused prior knowledge; namely, the self-similarity properties of network traffic. To increase the interpretability of the deep learning model, the temporal features of traffic sequences are extracted by FPK-Net through the combination of a one-dimensional convolutional neural network and an attention mechanism. In addition, the traffic self-similarity and attention-based long-short memory neural network are adapted to guide the prediction under various traffic sizes in future time periods. The accumulation of prior knowledge during training meaningfully guides the network traffic prediction, thus significantly improving the performance of the training model. In terms of the absolute coefficient of correction, the proposed model achieved values at least 10% higher than those of traditional statistical models. Thus, the reliability and superiority of the proposed model were illustrated in the article while the results demonstrated that the model is also interpretable.

## Figures and Tables

**Table 1:** Performance comparison of different methods on the dataset.

Models	MAE	MSE	RMSE	$R_{adapted}^{2}$
**HA**	0.447	0.341	0.584	0.604
**ARIMA**	0.615	0.573	0.757	0.509
**SVM**	0.391	0.339	0.583	0.677
**LSTM**	0.420	0.297	0.545	0.745
**CNN-LSTM-Attention**	0.387	0.286	0.535	0.750
**Transformer**	0.412	0.319	0.565	0.711
***FPK-Net***	0.369	0.259	0.509	0.769

**Table 2:** FPK-Net model prediction measures under different sliding window lengths.

Step	MAE	MSE	RMSE	$R_{adapted}^{2}$
**50**	0.390	0.284	0.533	0.753
**60**	0.388	0.282	0.531	0.758
**70**	0.384	0.275	0.525	0.763
**80**	0.382	0.274	0.524	0.765
**90**	0.382	0.274	0.524	0.765
**100**	0.372	0.269	0.519	0.768
**130**	0.369	0.259	0.509	0.769
**150**	0.373	0.261	0.510	0.764

## Data Availability

In this paper, the transit links of the WIDE network (AS2500) in Japan since February 2013 are selected as the data set for the experiment. The IP addresses shown in the dataset are anonymized using the prefix preservation method. In this paper, a total of 52,493 data were sampled from this dataset between 1 January 2020 and 31 December 2020, with a sampling interval of 10 min. The data download link is as follows: https://mawi.wide.ad.jp/~agurim/ (accessed on 1 February 2013).
